# Molecular Mechanisms of Pancreatic Stone Formation in Chronic Pancreatitis

**DOI:** 10.3389/fphys.2012.00415

**Published:** 2012-11-05

**Authors:** Shigeru B. H. Ko, Sakiko Azuma, Toshiyuki Yoshikawa, Akiko Yamamoto, Kazuhiro Kyokane, Minoru S. H. Ko, Hiroshi Ishiguro

**Affiliations:** ^1^Sakaguchi Laboratory, Department of Systems Medicine, Keio University School of MedicineTokyo, Japan; ^2^Department of Gastroenterology, National Center for Geriatrics and GerontologyObu, Japan; ^3^Yoshikawa ClinicShizuoka, Japan; ^4^Department of Human Nutrition, Nagoya University Graduate School of MedicineNagoya, Japan

**Keywords:** chronic pancreatitis, pancreatic stone formation, bicarbonate secretion, CFTR, cytoplasmic mislocalization

## Abstract

Chronic pancreatitis (CP) is a progressive inflammatory disease in which the pancreatic secretory parenchyma is destroyed and replaced by fibrosis. The presence of intraductal pancreatic stone(s) is important for the diagnosis of CP; however, the precise molecular mechanisms of pancreatic stone formation in CP were left largely unknown. Cystic fibrosis transmembrane conductance regulator (CFTR) is a chloride channel expressed in the apical plasma membrane of pancreatic duct cells and plays a central role in ${\text{HCO}}_{3}^{-}$ secretion. In previous studies, we have found that CFTR is largely mislocalized to the cytoplasm of pancreatic duct cells in all forms of CP and corticosteroids normalizes the localization of CFTR to the proper apical membrane at least in autoimmune pancreatitis. From these observations, we could conclude that the mislocalization of CFTR is a cause of protein plug formation in CP, subsequently resulting in pancreatic stone formation. Considering our observation that the mislocalization of CFTR also occurs in alcoholic or idiopathic CP, it is very likely that these pathological conditions can also be treated by corticosteroids, thereby preventing pancreatic stone formation in these patients. Further studies are definitely required to clarify these fundamental issues.

## Introduction

Chronic pancreatitis (CP) is a progressive inflammatory disease of the pancreas, and is characterized by pancreatic exocrine and endocrine dysfunction resulting from tissue damage caused by inflammation. The pancreatic exocrine gland is composed of two types of cells, duct cells and acinar cells. Duct cells secrete fluid and ${\text{HCO}}_{3}^{-}$ to neutralize gastric acid from the stomach. Acinar cells secrete digestive enzymes essential for the digestion of food. Regardless of the cause of pancreatitis, ${\text{HCO}}_{3}^{-}$ and digestive enzyme secretion are more or less compromised in all forms of chronic pancreatitis.

For a diagnosis of CP (Homma et al., 1997), it is essential to show the inflammation and destruction of the gland; however, it is often difficult to obtain pancreatic tissues due to the anatomy of the gland, except in cases of a pancreatic resection for malignant tumors. Another way to diagnose CP is to show exocrine pancreatic dysfunction. Pancreatic ductal dysfunction, especially a low ${\text{HCO}}_{3}^{-}$ concentration in the pancreatic juice, is the most important finding for the diagnosis of CP with mild or moderate exocrine dysfunction, since an impairment in ductal ${\text{HCO}}_{3}^{-}$ secretion is one of the earliest defects in CP (Freedman, 1998). For evaluating exocrine function, the secretin test was the only reliable test which can directly measure pancreatic ductal function (Ko et al., 2010) and acinar cell function separately; however, it became impossible to diagnose mild or moderate CP Functionally because the secretin test is no longer available in Japan due to the lack of supply of the clinical grade secretin. For that reason, the presence of pancreatic ductal stones became the most reliable diagnostic criterion for chronic pancreatitis.

Pancreatic stones are thought to be formed at first as protein plugs in pancreatic ducts in CP (Freedman, 1998). However, detailed molecular mechanisms of how pancreatic stones are formed in pancreatic ducts of CP still remain elusive. In previous studies, while elucidating the molecular mechanisms of aberrant ${\text{HCO}}_{3}^{-}$ transport in pancreatic ducts in chronic pancreatitis, we have found that the Cystic fibrosis transmembrane conductance regulator (CFTR) chloride channel, which plays a central role in ${\text{HCO}}_{3}^{-}$ transport in pancreatic ducts, is largely mislocalized in the cytoplasm of pancreatic duct cells in autoimmune pancreatitis (Ko et al., 2010). As the cytoplasmic mislocalization of CFTR has been observed in all other forms of CP (i.e., alcoholic, idiopathic, or obstructive), we concluded that the mislocalization of CFTR is a cause of pancreatic ductal dysfunction and subsequent pancreatic stone formation. This notion was further supported by the observation that corticosteroids, a potent anti-inflammatory drug, normalize the CFTR localization from the cytoplasm of pancreatic duct cells to the proper apical plasma membrane, and subsequently restored aberrant pancreatic ${\text{HCO}}_{3}^{-}$ secretion.

In this article, we propose that the cytoplasmic mislocalization of the CFTR in pancreatic duct cells is a cause of pancreatic stone formation in chronic pancreatitis. Currently, steroid treatment for autoimmune pancreatitis is the only established therapy to restore impaired pancreatic ductal function. It might be possible that pancreatic ductal dysfunction seen in alcoholic or idiopathic pancreatitis can also be cured by the administration of corticosteroid treatment. Further studies are definitely required to clarify these important issues in the treatment of chronic pancreatitis.

## ${\text{HCO}}_{3}^{-}$ Secretion from Pancreatic Duct Cells is Compromised in Chronic Pancreatitis

The normal pancreas secretes the most alkaline fluid among exocrine organs in humans (maximum ${\text{HCO}}_{3}^{-}$ concentration in pancreatic juice around 140 mM, pH ∼8.5) to neutralize acid from the stomach (Steward et al., 2005). In pancreatic ducts in chronic pancreatitis, it is well known that the alkalinization of pancreatic juice is impaired, and results in a low pH in secreted fluid. However, the precise molecular mechanisms of pancreatic ductal dysfunction remained elusive.

**Figure 1:**
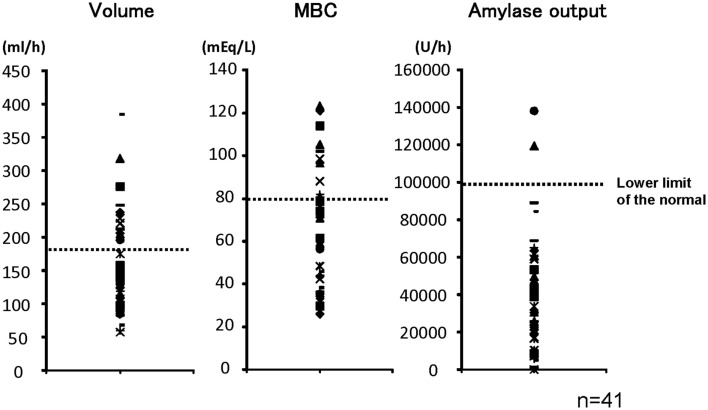
**Pancreatic exocrine functions are compromised in chronic pancreatitis**. Pancreatic exocrine functions of patients with alcoholic chronic pancreatitis were evaluated by a canonical secretin-stimulated exocrine function test. Pancreatic fluid volume (lower limit of normal, 183 ml/h), maximum bicarbonate ${\text{(HCO}}_{3}^{-})$ concentration in pancreatic juice (MBC: lower limit of normal, 80 mEq/l), and total amylase output (AO: lower limit of normal, 99,000 U/h), were all impaired in most of the patients with chronic pancreatitis (Ko et al., 2010).

Figure 1 shows pancreatic exocrine functions in patients with alcoholic chronic pancreatitis. Pancreatic fluid volume (V: lower limit of normal, 183 ml/h), maximum bicarbonate concentration (MBC) in pancreatic juice (lower limit of normal, 80 mEq/l), and total amylase output (AO: lower limit of normal, 99,000 U/h), were all impaired in alcoholic chronic pancreatitis. In a severe form of chronic pancreatitis, all three factors are impaired. In some cases with mild or moderate forms of chronic pancreatitis, fluid volume, and AO may stay within normal range; however, ${\text{HCO}}_{3}^{-}$ secretion is frequently impaired even in a mild form of the disease. Therefore direct measurement of maximum ${\text{HCO}}_{3}^{-}$ concentration in pancreatic juice was very valuable to diagnose mild or moderate forms of CP when the secretin test was available.

## Molecular Mechanism of Pancreatic ${\text{HCO}}_{3}^{-}$ Secretion from the Normal Duct Cells

**Figure 2:**
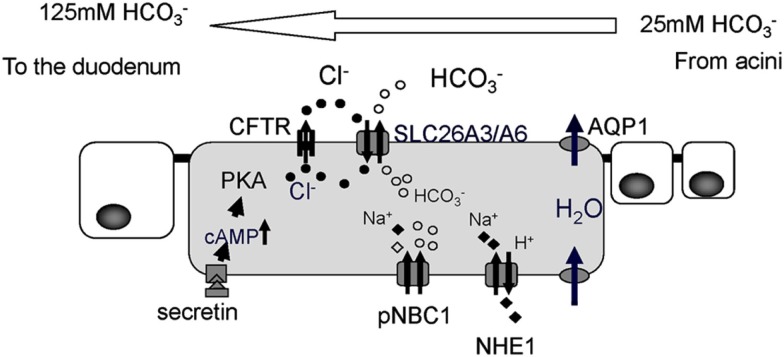
**Cellular mechanism proposed for ion and fluid secretion by the pancreatic duct epithelium**. The primary fluid secreted by acinar cells contains a large amount of digestive enzymes and 24 mM ${\text{HCO}}_{3}^{-}.$ Intracellular ${\text{HCO}}_{3}^{-}$ leaves the cell in exchange for Cl^−^, mediated by SLC26 anion exchangers. Cl^−^ is supplied to the lumen by a secretin-stimulated Cl^−^ channel, the Cystic fibrosis transmembrane conductance regulator (CFTR). For fluid movement across the epithelium, aquaporin (AQP) water channels are present at both the apical and basolateral membranes of smaller ducts in the rat (Furuya et al., 2002) and human pancreas (Burghardt et al., 2003). PKA, protein kinase A; pNBC1, pancreatic sodium bicarbonate cotransporter1; NHE1, sodium proton exchanger1.

The pancreatic duct epithelium is capable of secreting ${\text{HCO}}_{3}^{-}$ at a concentration of around 140 mM (Steward et al., 2005). Molecular mechanisms of how the pancreatic duct epithelium secretes such a high concentration of ${\text{HCO}}_{3}^{-}$ have long been examined (Figure 2). A primary fluid rich in digestive enzymes secreted from pancreatic acinar cells contains around 24 mM ${\text{HCO}}_{3}^{-}.$ The digestive hormone secretin was secreted from the endocrine cells in the duodenum when these cells were stimulated with gastric acid from the stomach. Secretin binds to its receptor on the basolateral membrane of pancreatic duct cells. An increase of intracellular cyclic AMP levels stimulates the CFTR chloride channel which is located at the apical plasma membrane of small pancreatic duct cells. Cl^−^ ions pass through the CFTR chloride channel into the luminal space of pancreatic ducts. ${\text{HCO}}_{3}^{-}$ was secreted from pancreatic duct cells in exchange for Cl^−^ absorption by an electrogenic anion exchanger, SLC26 transporters, expressed also at the apical plasma membrane of pancreatic ducts (Ko et al., 2002, 2004; Song et al., 2012). When Cl^−^ concentration in the juice becomes quite low, ${\text{HCO}}_{3}^{-}$ was secreted to the luminal space of pancreatic ducts through CFTR chloride channels (Ishiguro et al., 2009).

## CFTR Chloride Channel is Mislocalized to the Cytoplasm of Pancreatic Ducts in Chronic Pancreatitis

**Figure 3:**
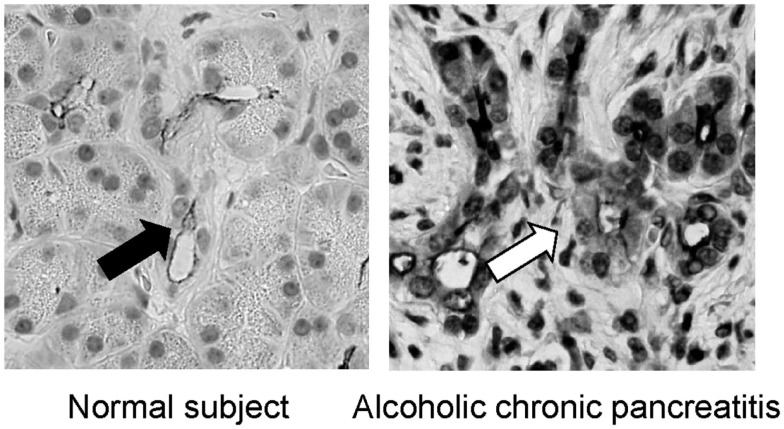
**Immnolocalization of CFTR in the pancreas of normal and chronic pancreatitis**. In the normal subjects, the CFTR chloride channel is exclusively localized in the apical plasma membrane of small pancreatic ducts (left panel). In contrast, the CFTR chloride channel is largely retained at the cytoplasm of pancreatic ducts and is not transported to the proper apical plasma membrane domain in alcoholic chronic pancreatitis (right panel; modified from Freedman, 1998).

It has been well known that the ${\text{HCO}}_{3}^{-}$ concentration in pancreatic juice is reduced in CP (Braganza et al., 2011). In previous studies, however, it was unclear why ${\text{HCO}}_{3}^{-}$ concentration in pancreatic juice in CP is low. It has been shown that the CFTR plays a most pivotal role in ${\text{HCO}}_{3}^{-}$ secretion in pancreatic duct cells. Thus, it is reasonable to consider whether or not the expression of CFTR is compromised in pancreatic duct cells in patients with chronic pancreatitis. To this end, we have examined the immunolocalization of the CFTR in these patients. In the normal pancreas the CFTR is expressed exclusively at the plasma membrane of small pancreatic ducts (Figure 3). However, in chronic pancreatitis, trafficking of the CFTR is largely compromised and the protein is largely retained at the cytoplasm of pancreatic ducts (Figure 3). CFTR plays a central role in ${\text{HCO}}_{3}^{-}$ secretion from pancreatic duct cells; therefore, the reduced CFTR expression at the apical membrane of pancreatic ducts should result in the low ${\text{HCO}}_{3}^{-}$ concentration seen in CP (Ko et al., 2011).

## Steroid Therapy Normalizes Cytoplasmic Mislocalization of CFTR to the Apical Plasma Membrane of Pancreatic Duct Cells in Autoimmune Pancreatitis and Recovers Aberrant ${\text{HCO}}_{3}^{-}$ Secretion in Chronic Pancreatitis

Autoimmune pancreatitis is one form of chronic pancreatitis. Autoimmunity is suspected to be its pathogenesis (Yoshida et al., 1995). Severe exocrine insufficiency has been reported in most of the cases (Ito et al., 2007; Frulloni et al., 2010; Ko et al., 2010). Steroid therapy improves the swelling of the gland and narrowing of the main pancreatic ducts, and reduces serum gamma globulin and immunoglobulin G subtype 4 (IgG4) levels.

**Figure 4:**
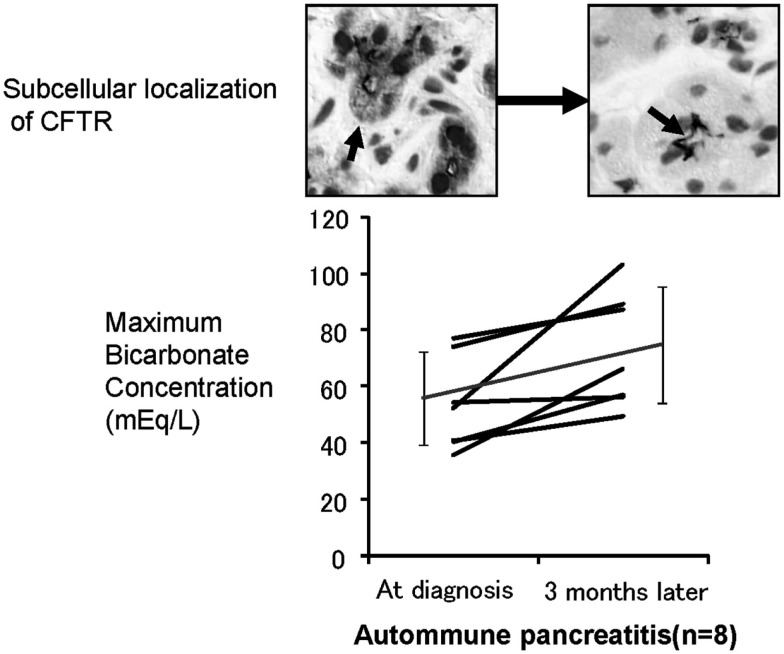
**Effects of corticosteroids on the localization of CFTR and pancreatic ductal dysfunction in autoimmune pancreatitis**. The lower panel shows the changes in ${\text{HCO}}_{3}^{-}$ concentrations in the pancreatic juice of autoimmune pancreatitis treated by corticosteroids. The ${\text{HCO}}_{3}^{-}$ concentration in pancreatic juice is quite low prior to treatment. However, 3 months of corticosteroid treatment significantly increased ${\text{HCO}}_{3}^{-}$ concentration in the pancreatic juice in autoimmune pancreatitis. The upper panel shows the changes of immunolocalization of the CFTR in human pancreatic ducts (modified from Freedman, 1998). Much of the CFTR proteins were retained at the cytoplasm of small pancreatic ducts and were not transported to the proper apical plasma membrane domain (left). Three months of corticosteroid treatment completely corrected the localization of the CFTR from the cytoplasm of pancreatic ducts to the apical membrane (right).

To further elucidate the role of CFTR mislocalization in the aberrant ductal ${\text{HCO}}_{3}^{-}$ secretion in chronic pancreatitis, we have examined histology (Mizuno et al., 2009) and exocrine functions of patients with autoimmune pancreatitis at the diagnosis and 3 months after maintenance steroid treatment (Ko et al., 2010). In autoimmune pancreatitis, ${\text{HCO}}_{3}^{-}$ concentration in pancreatic juice is remarkably reduced prior to treatment as well as in other forms of CP (Figure 4), whereas 3 months of steroid therapy restored the mislocalization of the CFTR to the proper apical plasma membrane and significantly improved the ${\text{HCO}}_{3}^{-}$ concentration in pancreatic juice (Figure 4). These data indicate that the mislocalization of the CFTR is a direct cause of a low ${\text{HCO}}_{3}^{-}$ concentration in pancreatic juice and that steroid treatment restores both the localization of CFTR and ${\text{HCO}}_{3}^{-}$ concentration in pancreatic juice in autoimmune pancreatitis.

## Pancreatic Ductal Dysfunction is not Recovered Spontaneously in Chronic Alcoholic Pancreatitis

**Figure 5:**
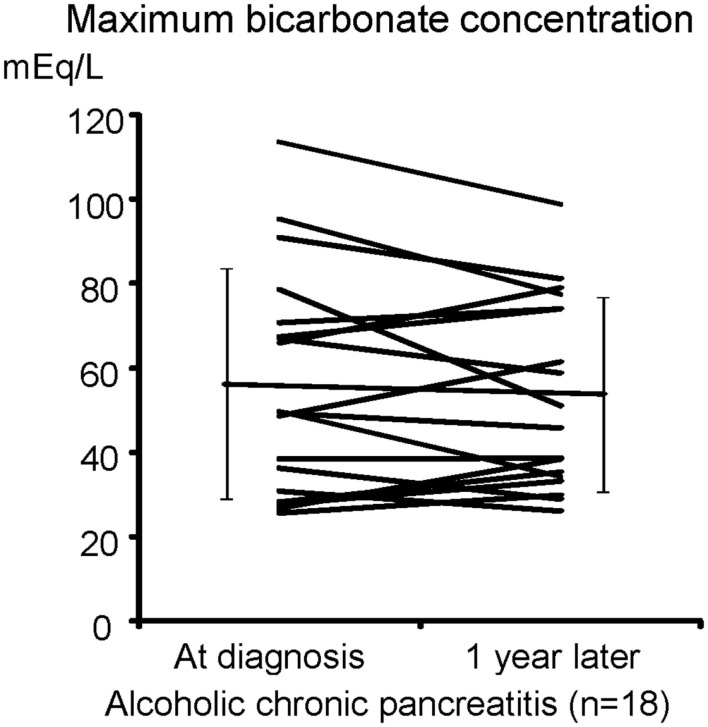
**${\text{HCO}}_{3}^{-}$ secretory defect is not spontaneously recovered in alcoholic pancreatitis**. The secretin-stimulated pancreatic exocrine test shows that a 1-year interval does not affect the ${\text{HCO}}_{3}^{-}$ secretory defect in alcoholic chronic pancreatitis.

We have shown that the mislocalization of the CFTR in the cytoplasm of pancreatic ducts is a possible cause of the low ${\text{HCO}}_{3}^{-}$ concentration found in pancreatic juice in chronic pancreatitis. If this proposed molecular mechanism is correct, one would speculate that the retargeting of the CFTR at the apical plasma membrane by the specific anti-inflammatory treatment will restore pancreatic ${\text{HCO}}_{3}^{-}$ secretion in chronic pancreatitis, and prevent pancreatic stone formation in chronic pancreatitis. Thus, we next examined ${\text{HCO}}_{3}^{-}$ concentrations in the juice of 18 cases of chronic alcoholic pancreatitis to see if pancreatic ductal dysfunction spontaneously recovers (Figure 5). As shown in Figure 5, a 1-year observation period did not affect the ${\text{HCO}}_{3}^{-}$ concentrations of pancreatic juice in chronic alcoholic pancreatitis without an active anti-inflammatory regimen, indicating that pancreatic ductal dysfunction does not improve spontaneously in chronic pancreatitis.

## Proposed Molecular Mechanism of Pancreatic Stone Formation in Chronic Pancreatitis

**Figure 6:**
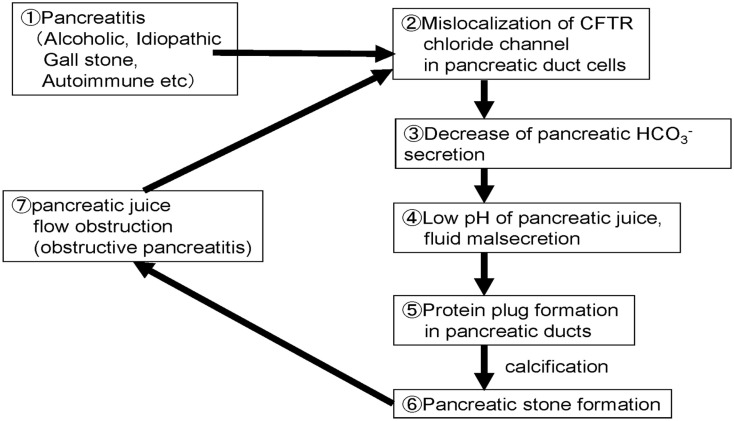
**Proposed cellular mechanisms of pancreatic stone formation in chronic pancreatitis**.

Figure 6 shows the possible steps/mechanisms for pancreatic stone formation in chronic pancreatitis.

Step 1: Chronic inflammation occurred in the pancreas by drinking, gallstone, or autoimmune mechanisms.
Step 2: Trafficking of membrane proteins such as the CFTR is compromised and the proteins are mislocalized to the cytoplasm of pancreatic ducts.
Step 3: Decrease of the CFTR expression at the plasma membrane results in a low ${\text{HCO}}_{3}^{-}$ concentration in pancreatic juice.
Step 4: Decrease of ${\text{HCO}}_{3}^{-}$ secretion further decreases the pH and volume of the fluid secreted by pancreatic ducts.
Step 5: Low fluid volume can result in the precipitation of digestive enzymes in pancreatic fluid and protein plugs are formed in pancreatic ducts in chronic pancreatitis.
Step 6: Protein plugs formed in pancreatic ducts disturb pancreatic juice outflow and protein plugs are calcified.
Step 7: Protein plugs and pancreatic stones obstruct pancreatic juice flow further and cause obstructive/upstream pancreatitis. Obstructive pancreatitis caused by protein plugs and stones in pancreatic ducts exacerbate the cytoplasmic mislocalization of CFTR, and then compromise pancreatic ductal dysfunction.

## Conclusions

Extensive research has revealed the molecular mechanisms of ion and fluid secretion in the physiological and pathological status (chronic pancreatitis) of the pancreas. Cytoplasmic mislocalization of the CFTR chloride channel results in aberrant ${\text{HCO}}_{3}^{-}$ secretion from the pancreatic ducts in chronic pancreatitis. Nonetheless, there is no cure for pancreatic ductal dysfunction in CP such as alcoholic or idiopathic pancreatitis. However, we have found for the first time that pancreatic ductal dysfunction of patients with autoimmune pancreatitis was partially reversed by the corticosteroid treatment. Further investigation to examine the effects of an anti-inflammatory regimen on pancreatic ductal dysfunction in other forms of Cp should eventually lead to the establishment of the treatment for chronic pancreatitis.

## Conflict of Interest Statement

The authors declare that the research was conducted in the absence of any commercial or financial relationships that could be construed as a potential conflict of interest.
